# Grey Zones, Ambiguous Zones: A Cogenetic and Dialogical Understanding of Sexual Consent

**DOI:** 10.1007/s12124-026-09975-2

**Published:** 2026-02-11

**Authors:** Juliane Obando, Angela Uchoa Branco, Luca Tateo

**Affiliations:** 1 Department of Human and School Development Psychology, University of Brasília, Brasília, Brazil; 2 Department of Special Needs Education, University of Oslo, Oslo, Norway

**Keywords:** Metacommunication, Grey Zones, Ambiguity, Cogenetic logic, Dialogical relations, Consent

## Abstract

In this article, we seek to articulate cogenetic and dialogical theory to understand the dynamics of ambiguities in human lives, using the phenomena of negotiating sexual relations as the empirical arena. We are interested in the factors that contribute to the maintenance of ambiguity in interpreting communicative messages in settings where experiences are characterized by ambivalence and ambiguity. We highlight ambiguity as a fundamental characteristic of social relations, arising from polysemy in the production and interpretation of signs. Most everyday social interactions express the constant need to negotiate meanings with others, and various “misunderstandings” (ambiguous “grey zones”) can therefore occur. Examples of interpretations were selected from individual interviews with nine participants, four young male and five female adults, aged 20–30, to analyze and discuss metacommunication processes that are expressed (or not) during sexual intercourse from reading a story. In this process of constructing meanings.

## Introduction

Ambiguity is an intrinsic characteristic of human communication. Its presence allows for continuous meaning negotiations that give rise to new affective-semiotic processes, which lie at the basis of dynamic semiosis. It confers flexibility and opens new venues for the permanent coconstruction of meanings as people interact/communicate with each other. Therefore, it is present in all kinds of communication, which can be conceived mainly as negotiation processes in which metacommunication plays a central role. In this article, we theoretically elaborate on this issue and illustrate its operation relative to the notion of consent, focusing on the topic of sexual consent between men and women. However, we believe similar processes may be involved in sexual consent between partners of different genders.

The grey zones of consent about sexual contact/relationship between heterosexual partners are a very relevant and controversial issue in the current society, often related to work and family relationships (Featherstone et al., 2024). They are directly linked to processes of creating and attributing meanings, and they have a significant, central role in the criminal justice systems, since determining if abuse or rape has happened is an unquestionable necessity. Therefore, to discuss the ambivalences and ambiguities present in the readings and interpretations of behaviors (verbal and non-verbal) related to pleasure, discomfort, rejection, and desire, it is also necessary to delve into the interpsychological and intrapsychological processes involved.

### The co-genesis of Signs and the Dynamics of Consent

An important characteristic of semiosis is that a complex of signs constitutes a dynamic totality based on *co-genetic logic* (Tateo, 2016). The production of a sign equals the production of a distinction in the undistinguished field or flow of events that immediately becomes a triadic system “A”-border-“non-A” (Tateo, 2024).

**Fig. 1:**
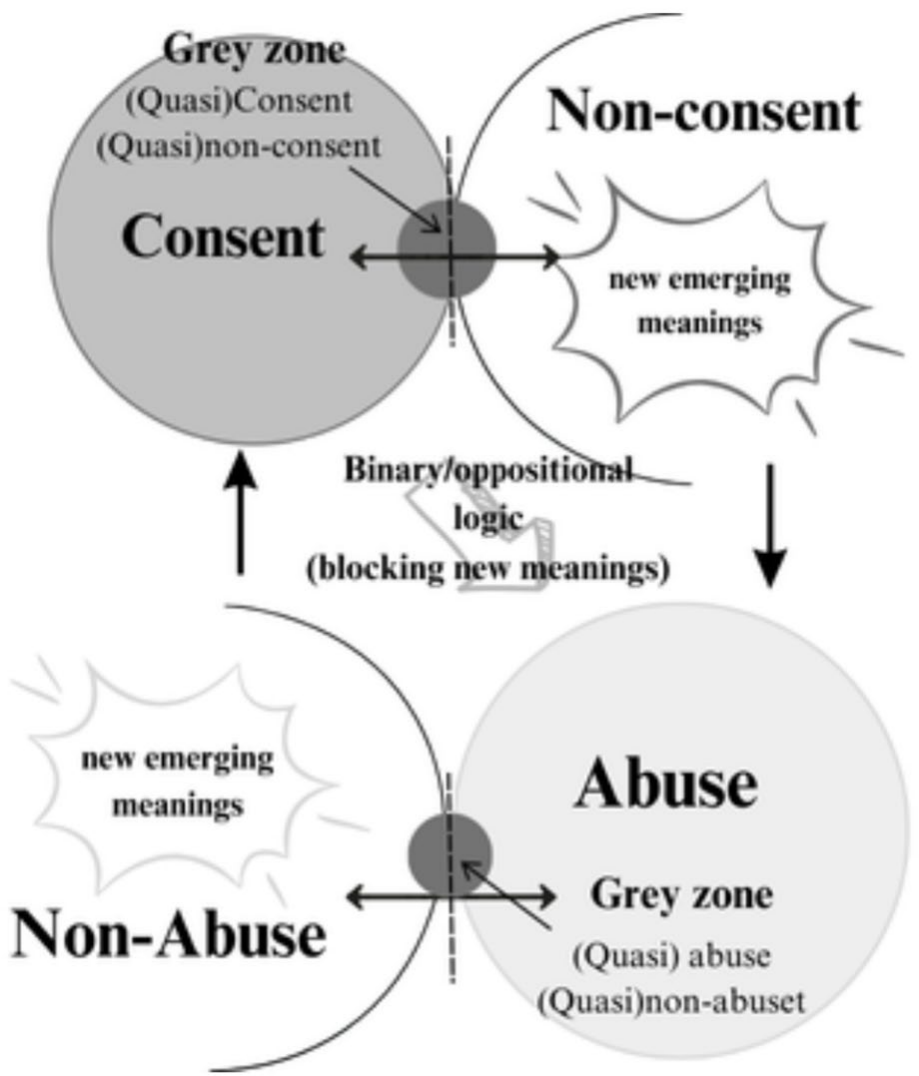
The dynamics of creating and assigning meanings related to consent/nonconsent and abuse/nonabuse

In strictly ontological terms, the complementary negation of a sign like “masculine” is not “feminine” (as it would be in common parlance) but “non-masculine”. This negative set includes, of course, the “feminine”, but also all the infinite possible forms that are not included in the current definition of “masculine”. The distinction between “masculine” and “non-masculine” is conventional and historically situated (Tateo, 2024). The border changes over time – for instance, the use of cosmetics that was once clearly considered “non-masculine” a few years ago has been progressively incorporated into the concept of “masculine.” In terms of meaning-making, the existence of co-genetic logic is very important because all those signs that exist on the buffer zone -the “quasi-A” signs- can, under certain conditions, become part of the closed-set definition of “A”. It is precisely in the buffer zone that the possibility of ambiguity emerges, together with that space of negotiation and aboutness that allows the emergence of new meanings. This logic guided the depiction of consent/nonconsent and abuse/nonabuse we present in Fig. 1.

Here, we assume that the grey zones of consent are directly linked to the processes of creating meanings. Figure 1 depicts, from a cogenetic perspective, what happens in the interpretation of meanings concerning the existence *versus* the non-existence of consent (in this case, relative to sexual consent or abuse). The opposite of consent is non-consent, not abuse. Non-consent, however, may give rise to abuse *versus* non-abuse, therefore creating a dynamic grey zone within which affective-semiotic processes generate constantly reconstructed meanings.

It is in this sense of a border space that we use the term Grey Zones of consent regarding sexual relations. While two people of opposite genders may think they share a similar understanding of actions and words that demonstrate consent or lack thereof in a specific cultural context, in practice, there can be significant differences in interpreting consent. This occurs, for example, in situations where “no” can be interpreted as “I don’t want to”, or “no, because I need to be convinced”, or as “I want to, but I don’t dare to speak out”. As we discuss later in this paper, it is precisely within these borders or Grey Zones that metacommunication, i.e., communication about communication, plays a fundamental role.

#### Consent in Contemporary Societies

In the book entitled “We Need to Talk About Consent”, authors Scarpati et al. (2024) define consent as a metric of what is casual and healthy (i.e., moral, practical, and legal) for sexual relations. Therefore, consent is often used as a determinant for understanding complex problems and for differentiating an experience between praise and harassment or sex and rape. However, the authors argue that simply identifying and discussing consent, although necessary, is not enough to encompass all the issues surrounding sexuality, pleasure, and sexual violence against women. Therefore, it is required to explore different terminologies or criteria to distinguish positive sexual interactions (consensual, non-violent, and desired) from aggressive, violent, criminal, or “grey” acts. An example of such “grey area” comes from women’s experience of harassment:

- [The woman’s] fear of being discredited or questioned also coexists with the desire to forget what happened. Often, the victim is so immersed in this denial that they do not even recognize that the experience was an assault or violation and have difficulty naming it as rape (Scarpati et al., 2024, p.17).

Scarpati et al. (2024) draw on feminist critical approaches to highlight the limitations of the reducible way we define consent. These approaches examine issues of inequality and the power imbalances that may exist in the negotiation and production of consent. In other words, critical approaches recognize that because consent is seen as an exclusive determinant for the existence of “acceptable” sexual encounter, in view of the inequalities present in human relations and between genders, it would become an empty concept and, consequently, confused with desire. That would happen because the concept is based on the understanding that people are in equal or similar conditions of knowledge, autonomy, and power to express their wishes accurately, as if that were possible. Differences between men and women, physical, cultural, and psychological, grant an asymmetry that cannot be denied.

Abdulali (2019) mentioned an experience of gang rape that he experienced and how the experience was socially interpreted: “I chose rape over death. Some people call this consent” (p. 62). Then, we can consider that one of the main factors that contributes to the maintenance of ambiguity in personal relationships would be the “myths of communication”, that is, assumptions in which the discourse would only reflect the subjective reality of the individual. This leads to the mistaken belief that observable behaviors, whether verbal or nonverbal, accurately reflect a person’s feelings, thoughts, and values, suggesting that there are no challenges in communication or interpretation (Angel, 2023, p. 165).

Values, beliefs, and social practices present in the cultural context in which the subject is inserted end up channeling, in different ways, desires and possible ways of experiencing sexuality. Although specific interest groups in a society tend to accept gender equality across various spheres of social life, the maintenance of cultural power hierarchies between men and women reflects archaic cultural meanings associated with gender relations (Madureira, 2010, 2016). So, it is worth investigating how men and women conceive and analyze possible indicators of consent, or abuse, in a sexual relationship.

Grey zones associated with consent to sexual practices can be described as situations, contexts, or experiences in which the attribution of meanings being coconstructed in the interaction between subjects is predominantly ambiguous. In other words, this occurs when, in the context of intersubjective verbal and nonverbal communication, different people read the same signs differently. Ambiguity thus arises as a communicative difficulty at both the intersubjective and the intrasubjective levels, where interpretative processes constantly occur (Sammut, Dannen & Moghaddam, 2013). As the issue of ambiguity is central to the discussion of this article’s topic, we will next address in some detail the key role ambiguity plays in human communication.

### Ambiguity in Everyday Life

Ambiguity reigns in human lives. For Ferreira et al. (2006), the study of ambiguity is relatively absent or underdeveloped in mainstream psychology due to its positivist nature. From a mainstream perspective, it is possible to see psychologists’ misguided attempts to reduce (or erase) ambiguity to a minimum. For example, in the psychological classifications and descriptions in the DSM (Diagnostic and Statistical Manual of Mental Disorders), the standardization and globalization of different psychological disorders allow no room for discussion of the important dimension of ambiguity.

At the same time, parallel studies on ambiguity, present in constructivist and semiotic-cultural approaches, are associated with the discursive context of ambivalence and the simultaneity of conflicting psychological processes involving affect and feelings (Abbey & Valsiner, 2005; Ferreira et al., 2006). These studies understand ambiguity as the core of interpersonal relationships, intersubjective processes, and the construction of meanings. By denying the existence of ambiguities or seeking to exclude them, we would miss an excellent opportunity to develop an in-depth understanding of various human processes, particularly the affective-semiotic processes occurring during communication. In fact, ambiguity is inherent to semiosis, which depends on the pleromatic realm of signs (Valsiner, 2024) involved when people try to make sense of each other’s meanings during social interactions.

The study of communicative processes emphasizes the multiplicity of simultaneous coexisting realities and significant signs in a social-symbolic sphere. Achieving agreements in communication relates to the fact that narrative and discourse are fundamentally based on affect. Culture is being constructed through processes of constructive internalization and externalization (Valsiner, 2014) on the part of the subjects involved, and it is through such processes that we create meanings and understand social phenomena (Brockmeier & Harré, 1997; Ferreira et al., 2006).

Culture and communication are fluid and dynamic; therefore, interpretation depends on the people involved, their history, affections, and the context in which they interact. Interpretations (such as consent, abuse, etc.) are constructed over the course of people’s lives, their communication and metacommunication interactions. Therefore, when we construct and attribute meanings, it is extremely important to be open to otherness, dialogue, and the contrast or tension between positions. As noted by Ferreira et al. (2006, p.10):

In a sense, meaning is created through a common understanding, but the original difference and asymmetry between these poles is a necessary condition for this bridge. In other words, meaning always depends on the interplay between equality and difference (…) This perspective establishes tension as a necessary condition for meaning. Dialogue, so commonly conceived as a way of establishing common understanding and cooperation, is also a matter of struggle, misunderstanding, and tension.

In other words, meaning is constructed through communicative interaction among subjects: the subject and the community/culture, and the self and the other. It emerges as a product while acting as a tool for interpsychological communication. As a result, processes of meaning construction occur at polysemic spaces, making it possible to envision infinite and diverse “truths”, interpretations, and plural readings of human phenomena.

### The Relevance of time

All meaning construction is important for highlighting the adaptive nature of affective-semiotic processes across the irreversibility of time (Valsiner, 2014). Human beings act in the present—facing the future that looks upon them—based on the reconstruction of their past experiences and anticipations or expectations regarding possible futures. Therefore, the meanings coconstructed in the present draw on elements from past situations and are also affected by expectations about future experiences. The context from which meaning emerges is unique and unrepeatable; however, the ambiguous nature of coconstructed meanings is necessary to allow room for negotiations and innovations arising from the inherent dialogical condition of communication.

### Ambiguity as the Core Feature in Communication

A certain degree of ambiguity is a fundamental feature of social relationships. It derives from the polysemy of signs’ production and interpretation. Imagine a social relationship in which people never tell a lie and always use precise words to describe their thoughts and actions. Such a relationship would soon come to a conflictual end. Indeed, the inherent “aboutness” of signs creates a space for modulation, negotiation, and dialogue, but also, of course, for potential misunderstanding and confusion. In pragmatic terms, communicative relationships are a collaborative enterprise (Grice, 1989), in the sense that the participants contribute to the production and interpretation of signs, although their goals and roles can differ.

**Fig. 2:**
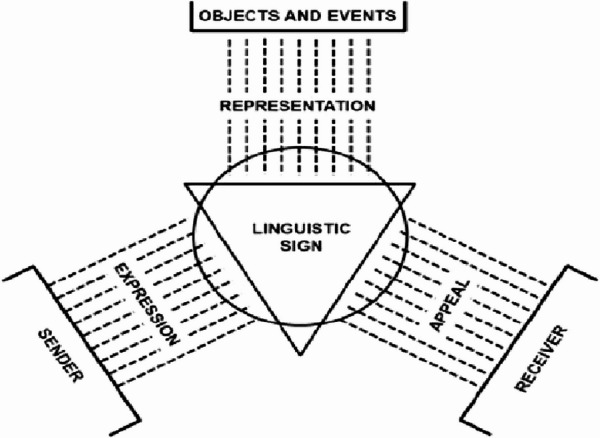
Buhler’s Organon Model

To better understand human communication as a creative and polysemic semiotic process, one can turn to authors who have accounted for the space of meaning negotiation inherent in the pragmatics and logic of communicative processes. One can begin with Karl Bühler (1879–1963), who developed a model of human communication known as the “Organon Model” (Fig. 2). In his model, one can identify three complementary functions in communicative actions: one related to the sender, one to the receiver, and one related to the object.

The starting point for communication is the sender’s personal, subjective understanding, which results in the constructed message. In doing so, the sender refers to an external situation that the receiver can also perceive as a Representation. The message encoded in signs has a certain impact, or Appeal, to the receiver, who must actively contribute to the interpretation. The non-overlapping of the sender and receiver perspectives implies that the same objective situation can never be understood in the same way by the participants to the interaction.

The key concept here is that the receiver is not just waiting for the message to be interpreted; they are also actively engaged in interpreting it. The receiver’s active contribution is based on a set of anticipations and expectations.

Communication requires the contribution of all participants, but this cooperation can take place at two different levels (Grice,^1989^). At the linguistic level, cooperation consists of fulfilling the mutual obligations in terms of valid utterances. For instance, using language that is understandable and appropriate to the situation.

At the social level, communication requires cooperation in reaching a common understanding. Thus, the sender may be cooperative at the linguistic level, but a discrepancy can exist between the sender’s and receiver’s communicative intentions at the social level. Let’s take, for instance, the following short dialogue:

- *Person A: Can you please pass the salt?*
- *Person B: Sure thing, I can!*

In this example, both participants produce valid utterances according to discussion rules. Neither person B violates the adjacent-pair question-answer sequence. Nevertheless, the reader is immediately aware that something’s out of place. Person B was indeed cooperative at the linguistic level, but Person A’s communicative intentions were not properly fulfilled. The sense of awkwardness does not arise from the message content, but from the fact that we have commonsense knowledge about how things should go in a typical “pass me the salt” dialogue script.

In the same line, let’s now imagine the following dialogue:

- *252 Person A: (Maybe) come for a ride some*.
- 253 day:.
- 254 (.) (.)
- *255 Person B: → I ↑will ↓see:. °when I ↑have*.
- *256 ↓time°*.

The second example is an excerpt from an actual conversational analysis study about sexual harassment (Tainio, 2003, p. 185). Person A is clearly uttering an invitation, but the reader can notice how the phrasing opens to multiple possible interpretations of the word “ride” that require some effort of decoding. Person B’s reply also exhibits a degree of polysemy, although the range of possible interpretations is constrained by nonverbal cues (a long pause before the answer; a tone modulation signaling that the person is uncomfortable). However, to make sense of the dialogue it is necessary that the participants somehow make assumptions about the background, the internal state, and the intentions of the communication partner, as well as referring to the “usual” way of communication in similar situations (common sense) (Rommetveit, 1998). This is not an exact science; it is rather an “educated guess” that we make several times a day when initiating a conversation. Most of the time, the intersubjective guess - the leap into another’s subjectivity (Rommetveit, 1998) – works because it is largely based on shared commonsense assumptions. Yet, every interaction implies a gap, an uncanny aspect *of interpretation*, and a certain degree of ambiguity. This is, of course, not a pathology of interpersonal relationships but rather the result of participants’ characteristics and the sensemaking process itself. The participants enter the interaction with their own histories, experiences, systems of values and beliefs, idiosyncrasies, expectations, and needs. These different ego-centered positions imply not only a risk of divergent interpretations but also the possibility of a richer look at the object of communication from multiple perspectives.

Moreover, the combination of signs that constitutes a message in everyday life communication is characterized by an “aboutness” of meaning. In the production and interpretation of signs, there is always a certain degree of *semiotic freedom* (Hoffmeyer, 2009) that is the lesser or greater number of possible interpretants that can be produced during the semiosis, whereas a zero-degree amounts to the absence of communication, and an infinite semiotic freedom equals the impossibility of interpretation. Semiosis requires a system of constraints or code to function. Human beings are capable of a wide variety of acts of semiotic freedom through different systems of signs.

For example, in a situation that requires exactness, such as a surgery, semiotic freedom must be limited, and there must be very few possible alternative interpretations of a sign, such as “close the wound”. If the same complex of signs is produced during a romantic situation, the degrees of semiotic freedom are greater as the use of metaphors serves to produce aboutness: “My beloved, let us fly together to the Moon and get impregnated with its peaceful silver joy…”.

Semiotic freedom thus varies according to the situation and the genre of discourse, in function of Grice’s (1989) social and linguistic levels of cooperation. Gregory Bateson conceptualized this feature in terms of digital and analogic communication (Bateson, 1972) as complementary forms of meaning-making.

### Ambivalence of Signs

Ambiguity, aboutness, and semiotic freedom are features of sign production and interpretation. These phenomena are best conceptualized by the notion of ambivalence. The term ambivalence was introduced by Eugen Bleuler (Bleuler, 1911/1950) as one of the four main symptoms of schizophrenia. He defined ambivalence as the “tendency to endow the most diverse psychisms with both a positive and negative indicator at the same time” (Bleuler, 1911/1950, p. 53). Also in affective terms, people can relate to the objects in a dual way (“A” and “non-A”). Conventionally, people also use dichotomies in the affective dimension: good and bad, pleasant and unpleasant, happy and sad, etc. Later, Freud (1955) considered ambivalence at the level of affects and ideas, which share the same energy and are developed in pairs, with one polarity displaced into the unconscious without being completely resolved.

At the level of ‘drive’ contents, the same object (e.g., a parent, a love partner, a car, a career achievement) can be invested in opposite drives, such as love and hate. The drives that are not socially acceptable (e.g., hate for children) are repressed but not eliminated, ready to come back in the form of creative (through sublimation) or disruptive (as neurosis or psychosis) outcomes. Interpersonal communication involves multiple levels— analogic and digital, social and linguistic—where ambivalence can exist.

The inherent ambivalence of human interactions is conceptualized by the *double bind theory* (Bateson, 1972), which describes an interpersonal situation in which a person is unable to resolve a communication dilemma due to the conflict between different logical levels (Bateson, 1972). The double bind creates an impossible situation from which it is difficult to escape, and repetition can lead to mental disorder (Bateson, 1972). The double bind communication is ambivalent at the levels of the message (opposite injunctions of different order and polarity); the power relationship (one participant must have some power of constriction over the other as well as an affective relation); the level of logic inconsistency (the injunctions lead to incompatible outcomes); the affective level (feelings of attraction and repulsion); and the level of meaning (impossibility to make sense of the situation and elaborate a solution). An example of double bind messages in a sexual encounter can be, “You know I love you!”, while physically acting violently. If the victim denies the truth of the first statement (“I love you”), they must/should admit the failure of the relationship. If the analogic message of the abusive behavior is denied (“he only beat me once”), the victim continues suffering the ambivalent personal relationship.

### Double Bind and Metacommunication

The only way to break a double bind-type situation and escape the meaning trap is to expose the ambiguity of the situation through a metacommunicative act. From a cultural psychology perspective, to escape the double bind, it is necessary to produce a sign at a higher hierarchical level that can defuse the trap by cancelling the effect of the previous conflicting signs (e.g., “love does not include abuse”). When it comes to romantic relationships and flirting, the distinction between the different communication levels of the content and of the relationship (Bateson, 1972) becomes crucial. Indeed, one of the situations in which a person becomes a victim of abuse is the case of the abuser who plays with the dimension of consent over the relationship (e.g., “I heard that she said no, but I know she meant yes”).

According to Abbey and Valsiner (2005), some ambivalence is inherent to semiosis and can usually be accepted (“it can be X…it can be Y”) or ignored (“but I don’t care”). Sometimes, people realize that what they think they know is not sufficient compared to what they know they do not understand. Then, ambivalence reaches higher levels, and the person can demolish the existing signs and start from scratch (“I don’t know anything”), or on the contrary can produce a “momentarily stable signs that mediate uncertainty by pre-controlling the meaning of a situation” (Abbey & Valsiner, 2005, p. 10) (“yes, it must be X!”).

A certain degree of ambivalence is therefore an attribute of interpersonal relationships (Bateson, 1972) and of every form of social organization (e.g., family, school, group of peers, political parties) based on a system of value-oriented practices that contain contradictory elements. Interpersonal relations occur within a cultural environment through various types of more or less conventional signs that require a common ground for effective communication. Nevertheless, a relationship based just on common ground would be unproductive, reducing the degree of semiotic freedom and potential novelty. In meaning-making,

- “some cracks need to be opened in the symbolic system, some inconsistencies need to emerge through which more spontaneous sign activities can unfold, and the other agencies can enter semiosis”

Without a degree of ambivalence, semiosis is impossible because there is no room for interpretation:

- “increasing and decreasing levels of ambivalence construct a self-perpetuating process of meaning construction and emergence of signs with a number of different conditions” (Abbey & Valsiner, 2005, p.6).

However, in specific meaning negotiation processes, such as sexual consent, misleading interpretations can cause significant psychological damage—or even trauma—to the person who was forced into the sexual activity without agreeing with it. The analysis of such situations requires not only the identification of subtle metacommunicative signs but also a keen ability or sensitivity to interpret the fuzzy dance involved in the complex body-and-mind negotiation processes.

### Metacommunication

Metacommunication has been defined as communication *about* communication (Leeds-Hurwitz, 1995). In psychology, we narrow down its meaning to relational metacommunication, that is, communication about the affective qualitative frame within which people in interaction coconstruct meanings. For example, when a teacher says “excellent job!” to a pupil, but her voice and facial expression are clearly ironic and depreciative, she creates a metacommunicative frame that makes the child feel terribly bad. This means that nonverbal signs are very powerful in helping people interpret each other’s intentions; consequently, to increase the chances of a likely, correct interpretation in communicative contexts, it is necessary to take into account the whole, complex dance of verbal and nonverbal signs exchanged by participants. Notwithstanding, this requires people to have a high degree of ability or sensitivity to the entire mix of signs that can be extremely subtle and culturally sensitive.

In cases involving sexual engagement, things can become even more difficult because sex often encompasses seduction games, where flirting strategies play a significant role. That is, not just observers may not be able appropriately interpret what is going on (see section on the empirical study), but also those in interaction may not be sure about what is happening. We will be back to this issue later.

### Inter and Intrapsychological Processes in the Border Zone of Consent

Metacommunication is, therefore, deeply ingrained in intersubjective processes between individuals (subjects) who negotiate a shared reality through interaction and communication. In this co-constructed reality, which can be understood as context, situation, interpretation, understanding, and/or reading about something or someone, the idiosyncratic experiences of each person seek to be reconciled with those of others through the negotiation of meanings. (Tateo & Marsico, 2021),

Interpsychological processes that occur during communication, then, contribute to the construction and maintenance of a common and shared reality, and affective metacommunicative frame, that integrates different meanings present for each individual, while also helping individuals to orient themselves and relate to one another. Thus, for communication to be effective, it is necessary to publicly express feelings, affections, memories, and other private issues, including the subjects’ intrasubjective processes (Sammut et al., 2013; Tateo & Marsico, 2021).

According to Sammut et al. (2013), most everyday social interactions express the constant need to negotiate meanings with one another, and various misunderstandings (ambiguities) arise because some meanings are sometimes interpreted as obvious or well-defined when, in reality, they cannot coherently reflect the social reality co-constructed between individuals. In other words, in our interactions and communication with the Other/World, we constantly negotiate different personal understandings (with a historical and affective basis) to establish a common context (reality).

Consider, for example, a flirtatious situation between two heterosexual young adults at a party. John sees Maria dancing alone and subtly approaches her to flirt. At first, the man understands that, because the young woman is at a party dancing alone, this would demonstrate her availability or interest in engaging with the people present. However, when he approaches Maria, she expresses discontent and a lack of interest in John. At first, John thinks that Maria is “playing hard to get” and persists for a while until she reiterates her position of disinterest, and John walks away.

This situation presents us with a typical scenario of misunderstanding and ambiguity in relation to a co-constructed reality - John’s attempt to flirt with Mary. The misunderstanding arises when specific, pre-established meanings, perceived as fundamental (John interpreting that Mary would be available because she was at the party alone), come into conflict with Mary’s behavior (Mary showing a lack of interest in John). A new co-constructed reality then emerges, in which John realizes that not every woman who is dancing alone is interested in a relationship.

In this sense, another concept that should be explored to understand the ambiguities present in interactions, relationships, and contexts is the concept of *border*, developed and explored in the studies by Marsico and colleagues (Tateo & Marsico, 2021). According to Tateo (2024), the term *“border”* is a constitutive part, fundamental to a cogenetic understanding of a phenomenon. In the human experience, the English word “border” is polysemic and can have different connotations, ranging from national, rigid, and concrete borders to intrapsychic, interpersonal, and flexible *borders* related to social distinctions. More specifically, according to Tateo and Marsico (2021):

- *Borders* can be material or immaterial, concrete or imaginary, well-defined or vague. They determine what is permissible or not within a circumscribed space. They also define what is known and what should be kept away as strange or dangerous. By drawing a border, we impose our norms on a physical or psychological territory (…) Borders mark the place of a difference, whether real or presumed. In this sense, a border is a space between things, and even if it apparently concerns mainly the physical environment, it is first and foremost a psychological process of constructing meaning. Borders delineate a space in the mind and in society. (p.711)

As discussed by Tateo and Marsico (2021), the construction of borders is therefore an act of semiosis, since signs emerge from an embodied, affective relationship, leading to the production of distinctions. This corresponds to an interpretative act, which generates a generalizing or associative element (Tateo, 2018). Thus, *borders* not only define what is passable or inaccessible, dividing order on one hand from chaos on the other, but also present people with possibilities for crossing them (Marsico, 2016). As Marsico et al. (2013) claim, three processes are necessary for the development of *borders* between individuals and society: the creation of meanings, the creation of distinctions, and the aggregation of value. They are built primarily to enable the individual to articulate, differentiate, and/or hierarchically integrate their relationship with the environment. In other words, borders guide and regulate how people relate to each other.

It is important to note that boundaries operate at different levels, such as intrapsychological (subjective), interpsychological (intersubjective), between social groups, and in material and psychological interactions, such as the fence that separates the private home from the public environment, delimiting the space we have access to and the one we should not invade. They act in a normative way, being cultivated as social norms that establish what should and should not be done.

The concept of the border understands that signs regulate the feelings, affections, and conduct of individuals. Thus, they draw a line between what is acceptable and what is not (Valsiner, 2014; Tateo & Marsico, 2021), based on messages disseminated through various cultural means (such as signs, films, laws, photographs, and magazines) in the social environment. It should also be noted that,

- The personal nature of the construction of meaning in self-regulation makes it possible, for example, for a person to know about an expectation or norm (or expect others to know about it) but ignore it or not use it (Tateo & Marsico, 2021, p. 717).

Why is the concept of borders significant to the present topic, sexual consent or abuse? We know that borders are not always clear-cut, let alone rigid (Marsico & Tateo, 2017), and this contributes to the persistence of ambiguity and polysemy. Ambiguity can be interpreted as a fundamental aspect of borders, as it creates a space for negotiation and dialogue, as well as for misunderstandings, conflicts, and confusions. In this way, *the Grey Zone between rejection and consent can be understood as a border space of ambiguity*. In other words, it corresponds to a border region that encompasses aspects and characteristics from both sides, making the interpretation of actions and the inference of intentions difficult to discern.

It is in this sense of a border space that we use the term *Grey Zones* of consent regarding sexual relations. While two people of opposite genders may think they share similar understandings of the actions and words that demonstrate consent or lack thereof in a specific cultural context, in practice, there can be significant differences in the interpretation of consent. This occurs, for example, in situations where “no” can be interpreted as “I don’t want to”, or “no, because I need to be convinced”, or as “I want to, but I don’t have the courage to speak out”.

### Ambivalence of Signs on the Border Zone of Consent: Illustrations from an Empirical Study

To illustrate the “Grey Zones” related to negotiations of sexual relationships, we extracted excerpts from the statements of participants in the doctoral research of the first author of this article, entitled “The grey zones of consent: sexual violence from the perspectives of young, straight, cisgender men and women.” The research used three procedures: discussions about the display of images, an edited video excerpt, and a short story—all materials related to situations of ambiguity regarding the possible interpretations of the scenes in terms of consent or its absence. The research was conducted online with 10 young adults—5 women and 5 men—aged 20 to 30 from different Brazilian states. In this section, we selected a few participants’ narratives from their evaluations of the short story we asked them to read during individual semi-structured interviews.

### The target material 

The story was about Carla and Rogério, based on an account from Grigoriadis’s book (2018). The story, in short, showed a couple who met through a social network and then met in person at a bar. Leaving the bar, Rogério ends up luring Carla to his apartment, and from there, he does everything he can to convince her to have sex with him, while Carla gives him ambiguous signals until he proceeds to have sex with her anyway.

It is important to emphasize that the story was written descriptively and objectively, without providing nonverbal cues or exploring the possible feelings, affections, or emotions of those involved. The story did not provide complete access to their verbal and nonverbal actions, reactions, facial and body expressions, or signs indicating pain, discomfort, desire, or pleasure. Therefore, participants had freedom to create and imagine what happened in the story, which allowed the researcher to have some access to participants’ beliefs, values, and feelings as they interpreted whether Carla consented to the sexual act. Participants had to imagine what happened and base their analyses on subjective evaluations.

In the interview with Anezka (participants’ names are fictitious), for example, various ambiguities emerged that made her interpretation difficult:

- *I can’t say*. I’d have to ask Carla (…) because she said she didn’t want to… I don’t think it was consensual, no, because when someone gets overwhelmed by exhaustion, like, when the other person has already gotten physical, they might be scared, they might have difficulty setting limits, so *I don’t think it was consensual*,* no.* (Anezka)

According to Anezka, Carla’s opinion or perspective on what happened to her would determine whether consent was present, but her perspective was absent from the story. One of the interesting points the participant raised was the role of Carla’s subjective processes, such as beliefs, feelings, expectations, projection, and/or associations, in determining whether violence occurred. For example, although the story does not provide clear information indicating that Carla was afraid and had difficulty setting limits in her interactions with Roberto, Anezka raised this interpretation during the interview. The participant was thus absolutely right. However, the main point is that even when we can observe interactions, we would base our interpretation on specific signs (verbal and nonverbal) of metacommunication to assess the presence of consent! But what if such signs are not clear? Or the girl herself cannot or does not express how she is feeling about it, due to fear or even mixed feelings?

Beyond doubt, the whole thing is too difficult to interpret, and each participant then interpreted the situation according to their values and previous experiences. In other words, past experiences—previous contact with certain situations—may guide a person’s interpretation and positioning concerning the interpretation of highly complex social/sexual situations in the present.

It is also noteworthy to mention that Anezka, by emphasizing the motivations, feelings, and affections of the girl in the story, raises the possibility that Carla had consented to have sexual relations with Rogério, even after verbally expressing “no” to him, due to a change of mind/interest/desire on her part. After all, saying “no” can be part of a game of seduction, which often happens in affective-sexual interactions between two partners. In other words, there are multiple interpretations possible. However, this same ambiguous situation led Isaac to develop a very different interpretation. Although the participant also reported the text’s lack of clarity regarding consent or otherwise regarding the sexual activity, he said:

- I don’t think so [that she was abused] because of that little word [written in the report], *“no longer reacted*.” I don’t know… “no longer reacted” *implies that she stayed still*. (Isaac)

According to him, the lack of reaction mentioned in the story—translated and interpreted as verbal silence and the absence of physical resistance or any movement suggesting denial on Carla’s part—was sufficient to lead Isaac to the conclusion that the girl had consented. The absence of further reaction directed his reading toward the occurrence of a consensual relationship. “Staying still” was understood as “not reacting”, and this was interpreted as consent.

Nevertheless, there was a vast range of behavioral and contextual possibilities within the concept of “non-reaction” to a sexual advance. The range of all the potential meanings, of course, is constrained by the degrees of semiotic freedom that selectively allow only a few actual interpretations. The role of culture is to selectively channel the range of interpretations that can be actualized. For example, Carla might not have “reacted” to Rogério’s sexual advances. However, nothing in the story suggested that she did not say or do anything during the act, such as expressing with her face or eyes her desire for the relationship to end as quickly as possible, or even for it never to occur at all.

By interpreting the act of being “still” literally, Isaac considered it merely a radical inaction. He failed to consider the occurrence of possible intrasubjective factors or states experienced by the character. Furthermore, the signs described in the story, presented by Carla before the relationship, such as (a) *refusing* to sit on Rogério’s lap and (b) saying they would have sex the *next time* they saw each other, were not considered or given as relevant to the isaac’s definition of what happened (violence or not).

### Final Remarks: the Central Role of Metacommunication

In this article, we aimed to theoretically investigate the issue of ambiguity, inviting the reader to reflect on the difficulties associated with interpreting some sexual relations as either consented to or not consented to or characterized as abuse. To increase our ability as a just society to distinguish among all possible interpretations, we need to invest in deeper investigation of the phenomena, taking into account cultural, intersubjective, and subjective levels of analysis (Buhler et al., 1967; Valsiner, 2021). Among all factors to be considered, it is crucial to recognize how traditional female and male socialization processes can further foster the occurrence of ambiguities, distorted interpretations, doubts, and uncertainties that are present in some situations of sexual violence against women (Grigoriadis, 2017). In this sense, co-genetic studies of boundary phenomena and the understanding of dialogism in human interactions, as well as the construction and attribution of signs in metacommunication among subjects, present themselves as tools with great analytical potential (Marsico & Varzi, 2015). Such studies can also be valuable for exploring the existence of ambiguity (grey zones) in other spheres involving the dynamic complexity of human interactional processes.

There are numerous difficulties and limitations in interpreting interactional and subjective phenomena, especially when we consider that affective-semiotic experiences are expressed not only through speech but particularly through people’s bodies in complex ways. In other words, there is no way to fully assess complex phenomena such as consent. In the case of experiences in intimate and sexual relationships, the level of ambiguity becomes a predominant aspect, since in this domain, ambiguities are inherent to what has come to be called “games of seduction.” To contribute to this sensitive issue, we suggest further study and analysis of the phenomenon of metacommunication, as developing people’s—particularly males’—sensitivity to subtle nonverbal signs is of fundamental relevance. In short, to be aware of the essential role played by metacommunication in human interactions, especially when sex is involved, can make a substantial difference. In many cases, for instance, men end up raping women (from the female perspective) because they are not able to recognize such signs. Therefore, we hope that investigating metacommunication and bringing to social attention this issue, as well as matters of ethics, empathy, and respect in human interactions, can contribute to reducing violence against women.

## Data Availability

No datasets were generated or analysed during the current study.
